# Pericardial injury from chest compression: a case report of incidental release of cardiac tamponade

**DOI:** 10.1186/s40560-018-0325-5

**Published:** 2018-08-28

**Authors:** Shigeaki Aoyagi, Tomokazu Kosuga, Kumiko Wada, Shin-ichi Nata, Hiroshi Yasunaga

**Affiliations:** Department of Cardiovascular Surgery, St. Mary’s Hospital, 422 Tsubukuhonmachi, Kurume, 830-8543 Japan

**Keywords:** Aortic dissection, Cardiac tamponade, Cardiopulmonary arrest, Cardiopulmonary resuscitation, Chest compression, Pericardial injury

## Abstract

**Background:**

Although chest compression is a standard technique in cardiopulmonary resuscitation, it is well recognized that manual chest compression causes various internal injuries, of which major injuries are often fatal. Similarly, when cardiac tamponade occurs in patients with type A acute aortic dissection, many patients die before reaching the hospital. We report a rare case in which chest compressions caused pericardial laceration that may have inadvertently played a life-saving role in releasing cardiac tamponade induced by acute aortic dissection.

**Case presentation:**

A 67-year-old woman developed cardiac arrest soon after complaining of epigastric pain, and after successful resuscitation by manual chest compression, she was transferred to our hospital. On arrival, the patient was 14 on the Glasgow Coma Scale. An ECG showed a normal sinus rhythm, and no arrhythmias or signs of myocardial ischemia were observed. A chest X-ray revealed left pleural effusion, while cardiomegaly and pneumothorax were not identified. Computed tomography revealed type A aortic dissection, mild pericardial effusion, and massive left pleural effusion. No pulmonary embolus was found on the CT. After drainage of bloody effusion from the left pleural space, an emergency operation was begun. During surgery, a pericardial laceration with communication to the left pleural space and a hemothorax were found; however, no cardiac injury was identified. No other intra-thoracic injuries or rupture of the aortic dissection causing the hemothorax were detected. Hemiarch replacement was performed without difficulty, but the patient died of multi-organ failure 30 days after surgery.

**Conclusions:**

We report a case of pericardial injury without skeletal fracture caused by chest compression. The pericardial laceration may have inadvertently served to release the cardiac tamponade induced by the acute aortic dissection, resulting in the hemothorax, and provided time to receive surgery.

## Background

Chest compression is an effective cardiopulmonary resuscitation (CPR) technique for patients who develop cardiac arrest. However, various internal injuries caused by chest compression are well recognized, and these major intra-thoracic injuries are often fatal [1, 2]. On the other hand, aortic dissection is a catastrophic pathology, and cardiac tamponade is its common and lethal complication [3, 4]. For cardiac tamponade, pericardiocentesis or pericardial drainage is an effective treatment and occasionally a life-saving procedure [3].

In this paper, we report a rare case of these two phenomena working synergistically, in which pericardial injury caused by chest compression may have inadvertently played a life-saving role in releasing cardiac tamponade due to aortic dissection, thus providing extra time to receive surgical treatment.

## Case presentation

**Fig. 1:**
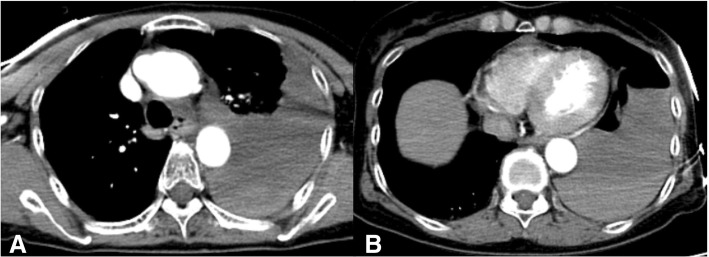
Preoperative CTs. CTs showing thrombosed type A aortic dissection with ulcer-like projection in the proximal ascending aorta, massive left pleural effusion (**a**), and mild pericardial effusion (**b**)

**Fig. 2:**
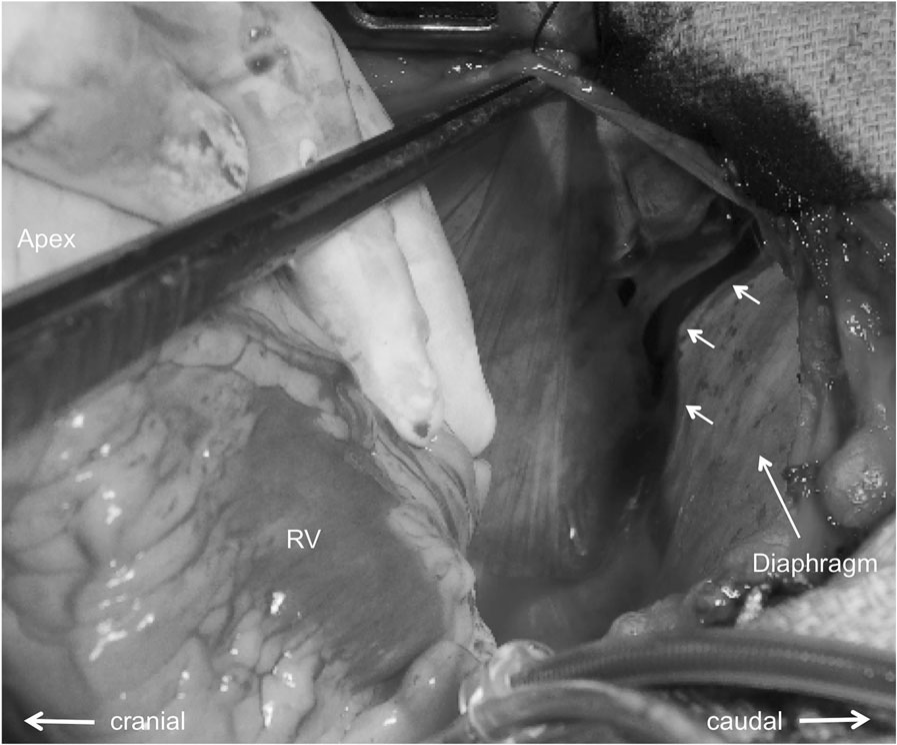
Intraoperative photograph. Photograph showing the large laceration (arrows) of the pericardium at the phrenico-pleural junction communicating to the left pleural space. RV right ventricle

A 67-year-old woman, in good health other than systemic hypertension, lost consciousness soon after complaining of severe epigastric pain at her workplace. The ambulance crew found the patient in cardiopulmonary arrest and paramedics immediately started CPR by manual chest compressions; return of spontaneous circulation and recovery of consciousness occurred 4 min later. On arrival at the emergency room, the patient’s level of consciousness was 14 on the Glasgow Coma Scale, blood pressure was 102/74 mmHg, and pulse rate was 103/min. No cardiac murmur was detected, but vesicular breath sounds were moderately diminished in the left lung field. Cardiac enzyme studies were not consistent with a diagnosis of myocardial infarction. An ECG showed a normal sinus rhythm, and no arrhythmias or signs of myocardial ischemia were observed. A chest X-ray revealed massive left pleural effusion with no right pleural effusion, while cardiomegaly and pneumothorax were not identified. Transthoracic echocardiography demonstrated normally functioning ventricles and valves, and mild pericardial effusion. Computed tomography (CT) showed a type A acute aortic dissection (AAD) with thrombotic occlusion of the false lumen and an ulcer-like projection in the proximal arch, along with mild pericardial effusion and massive left pleural effusion (Fig. 1). Occlusion of the branch vessels of the aortic arch and pulmonary emboli were not detected. Immediately after the CT, the patient fell into circulatory collapse. After drainage of bloody effusion from the left pleural space, an emergency operation was begun through a median sternotomy. No sternal fracture and bleeding in the mediastinum were found. When the pericardium was opened, a small amount of bloody effusion was present, but cardiac injury was not observed. In addition, a large laceration (10 cm) was found in the left posterolateral pericardium at the phrenico-pleural junction, through which the pericardial cavity communicated to the left pleural space (Fig. 2). Neither injuries of other intra-thoracic organs such as the lung, vessels, or chest wall causing the hemothorax nor external rupture of the dissection were detected. Under cardiopulmonary bypass and cardiac arrest, the aorta was opened. The dissection with the thrombosed false lumen extended from the aortic root to the aortic arch. A primary tear was present on the lesser curvature of the proximal arch. Hemiarch replacement including the primary tear was performed without difficulty, but a large amount of inotropic agents was necessary for weaning off cardiopulmonary bypass. The postoperative course was complicated with severe low cardiac output syndrome, and the patient eventually died of multi-organ failure on postoperative day 30. An autopsy was not permitted.

## Discussion

Effective chest compression remains the cornerstone of successful CPR, and manual chest compression is still a standard procedure [5]. However, a variety of internal injuries to intra-thoracic organs as well as abdominal viscera has been recognized [6]. According to a previous study [2], pericardial injury developed in nine (8.9%) out of 101 patients treated with CPR. Generally, pericardial injury develops together with rib or sternal fracture [2]. During repetitive compressions of the chest wall, displaced fragments of rib or sternal fracture directly injure the pericardium and the heart [5, 7]. However, pericardial injuries without a thoracic wall injury from chest compression have also been noted [8, 9]. In our patient, no rib or sternal fragment was present around the pericardial laceration. Furthermore, no injuries to intra-thoracic organs and rupture of the dissection causing the hemothorax were found during surgery. These findings indicate that the cardiac arrest occurred from cardiac tamponade induced by the AAD, after which vigorous chest compressions likely caused the pericardial laceration that drained the cardiac tamponade into the left pleural space, resulting in the hemothorax. In fact, hemothorax, not cardiac tamponade, has often been reported in patients with traumatic cardiac rupture who also have pericardial laceration [10].

Cardiac tamponade is the leading cause of mortality of type A AAD [3], and in-hospital mortality from type A AAD with cardiac tamponade was reported to be 54%, which was more than twice the mortality rate without cardiac tamponade [4]. In addition, when cardiac tamponade occurs in patients with type A AAD, many patients die before reaching the hospital and before a diagnosis is made. In most cases of cardiac tamponade, pericardiocentesis or pericardial drainage is not only an effective treatment but also a life-saving procedure; however, in cardiac tamponade induced by AAD, these procedures are normally contraindicated (excluding controlled drainage of a small amount of pericardial effusion) because of abrupt and excessive elevation of blood pressure leading to rupture or more extension of dissection [4]. Thus, pericardial laceration from chest compression provides a high potential for unfavorable outcome. However, in our patient, the pericardial laceration may have played an incidental life-saving role in releasing the cardiac tamponade, which led to successful CPR, and providing time to receive surgical treatment. Even though the risk for internal injuries leading to unfavorable outcome is present with proper CPR procedures, CPR should be promptly started for any patients who develop cardiopulmonary arrest because it is no question that immediate CPR can increase the chance of recovery. Similarly, Kan et al. [8] reported a case of cardiac tamponade due to postinfarction left ventricular rupture, which was relieved by a pericardial tear from resuscitation and concluded that the pericardial tear allowed for timely surgery. In addition, Okuda et al. [9] presented two autopsy cases of a pericardial tear as a consequence of CPR involving chest compressions in type A AAD with hemopericardium. Two pathologic findings—the presence of cardiac tamponade before CPR and an injury in the left posterolateral pericardium—were found in all four of these patients including the present patient. Considering these findings, high wall tension on the pericardium resulting from a steep rise in intra-pericardial pressure in the acute cardiac tamponade, along with shearing force generated on the junction of the two areas of the pericardium (the diaphragmatic and pleural pericardium) moving in different directions during chest wall compressions, may be the mechanism of the laceration in the posterolateral pericardium without skeletal fracture in this patient [9].

## Conclusions

We report a rare case of pericardial injury caused by chest compression. The pericardial laceration may have inadvertently served to release the cardiac tamponade induced by the AAD, resulting in the hemothorax, and therefore provided time to receive surgical treatment.

## Abbreviations

AAD: Acute aortic dissection
CPR: Cardiopulmonary resuscitation
CT: Computed tomography
ECG: Electrocardiogram

## References

[1] Buschmann CT, Tsokos M (2009). Frequent and rare complications of resuscitation attempts. Intensive Care Med.

[2] Miller AC, Rosati SF, Suffredini AF, Schrump DS (2014). A systematic review and pooled analysis of CPR-associated cardiovascular and thoracic injuries. Resuscitation.

[3] Hayashi T, Tsukube T, Yamashita T, Haraguchi T, Matsukawa R, Kozawa S (2012). Impact of controlled pericardial drainage on critical cardiac tamponade with acute type A aortic dissection. Circulation.

[4] Gilon D, Mehta RH, Oh JK, Januzzi JL, Bossone E, Cooper JV (2009). Characteristics and in-hospital outcomes of patients with cardiac tamponade complicating type A acute aortic dissection. Am J Cardiol.

[5] Rabl W, Baubin M, Broinger G, Scheithauer R (1996). Serious complications from active compression-decompression cardiopulmonary resuscitation. Int J Legal Med.

[6] Rudinska LI, Hejna P, Ihnat P, Tomaskova H, Smatanova M, Dvoracek I (2016). Intra-thoracic injuries associated with cardiopulmonary resuscitation – frequency and serious. Resuscitation.

[7] Agdal N, Jorgensen TG (1973). Penetrating laceration of the pericardium and myocardium and myocardial rupture following closed-chest cardiac massage. Acta Med Scand.

[8] Kan C-B, Chu I-T, Chang R-Y, Chang J-P (2010). Postinfarction left ventricular rupture salvaged by resuscitation induced pericard tear. Ann Thorac Surg.

[9] Okuda T, Takanari H, Shiotani S, Hayakawa H, Ohno Y, Fowler DR (2015). Pericardial tear as a consequence of cardiopulmonary resuscitation (CPR) involving chest compression: a report of two cases of acute type A aortic dissection with hemopericardium. Lega Med.

[10] Oizumi H, Suzuki K, Hoshino H, Tatsumori T, Ichonokawa H (2016). A case report: hemothorax caused by rupture of the left atrial appendage. Surg Case Rep.

